# Viable mouse gene ablations that robustly alter brain Aβ levels are rare

**DOI:** 10.1186/1471-2202-11-143

**Published:** 2010-11-05

**Authors:** Jeremy H Toyn, Xu-Alan Lin, Mark W Thompson, Valerie Guss, Jere E Meredith, Sethu Sankaranarayanan, Nestor Barrezueta, John Corradi, Antara Majumdar, Daniel L Small, Melissa Hansard, Thomas Lanthorn, Ryan S Westphal, Charles F Albright

**Affiliations:** 1 Neuroscience Biology, Bristol-Myers Squibb Research and Development, 5 Research Parkway, Wallingford, Connecticut 06492, USA; 2 Applied Genomics, Bristol-Myers Squibb Research and Development, 5 Research Parkway, Wallingford, Connecticut 06492, USA; 3 Global Biometric Sciences, Bristol-Myers Squibb Research and Development, 5 Research Parkway, Wallingford, Connecticut 06492, USA; 4 Lexicon Pharmaceuticals, Department of Neurology, 8800 Technology Forest Place, The Woodlands, Texas 77381, USA; 5 MDS Pharma Services, 22011 30th Dr SE, Bothell, WA 98021, USA

## Abstract

**Background:**

Accumulation of amyloid-β (Aβ) peptide in the brain is thought to play a key pathological role in Alzheimer's disease. Many pharmacological targets have therefore been proposed based upon the biochemistry of Aβ, but not all are equally tractable for drug discovery.

**Results:**

To search for novel targets that affect brain Aβ without causing toxicity, we screened mouse brain samples from 1930 novel gene knock-out (KO) strains, representing 1926 genes, using Aβ ELISA assays. Although robust Aβ lowering was readily apparent in brains from a BACE1 KO strain, none of the novel strains exhibited robust decreases in brain Aβ, including a GPR3 KO strain, which had previously been proposed as an Aβ target. However, significantly increased Aβ was observed in brain samples from two KO strains, corresponding to genes encoding the glycosylphosphatidylinositol mannosyl transferase PIGZ and quinolinate phosphoribosyltransferase (QPRT).

**Conclusions:**

Thus, gene ablations that are permissive for mouse survival and that also have a robust effect on Aβ levels in the brain are rare.

## Background

The amyloid hypothesis states that Alzheimer' disease (AD) is caused by accumulation of toxic forms of the amyloid-β (Aβ) peptide in the brain. Aβ is a secreted peptide formed through consecutive proteolytic cleavages of the amyloid precursor protein (APP) by the β-site APP cleaving enzyme (BACE1), which releases the N-terminal end of Aβ, and γ-secretase, which releases a range of Aβ C-terminal ends resulting in Aβ peptides of typically 37-42 amino acids in length. The predominant form of Aβ has 40 amino acid residues, and is denoted Aβ40, whereas the disease-associated Aβ42 has two additional C-terminal residues [1]. On the basis of the biochemistry and pathology of Aβ, many molecular targets have been proposed for inhibition of Aβ accumulation, aggregation, or the toxic effects of Aβ [2-5]. Thus, Aβ formation can be targeted directly via inhibition of BACE1 or γ-secretase, or indirectly via inhibition of pathways that regulate the activity of these proteases. Indirect regulation of BACE1 involves a particularly wide range of mechanisms, recently reviewed in detail by Hunt and Turner [6], and of potential pathological relevance because of the increased BACE1 activity observed in the AD brain [7-10]. In brief, BACE1 activity can be regulated through a variety of molecular targets involved in cytokine signaling [11,12], hypoxia [13,14], oxidative stress [15], energy deprivation [16], intracellular trafficking and maturation [17-19], and glycosylphosphatidylinositol (GPI) anchor metabolism [20-22]. Indirect targets have also been reported to regulate γ-secretase activity, including GSK3α [23], Rac1 GTPase [24], casein kinase I [25] and the G-protein coupled receptor GPR3 [26]. In addition, competition between the proteasome and γ-secretase for the C-terminal BACE1-derived APP processing intermediate can affect Aβ levels in cell cultures [27].

APP itself is a direct target of small molecule modulators of Aβ production [28,29], and can be targeted indirectly via the prolyl isomerase Pin1 [30], sphingolipid metabolism [31], reticulon/Nogo proteins [32,33], Nogo-like LRRTM3 [34], sorLA [35] and membrane microdomain switching [36]. APP-mediated changes in Aβ can also result from increased cleavage of the non-amyloidogenic α-site of APP, thus competing with BACE1 for the available APP substrate, as reviewed by Fahrenholz [37]. In brief, metalloproteases such as ADAM10 [38] carry out α-site cleavage, which can be activated via multiple targets, including retinoic acid receptor [39,40] liver-X-receptor, muscarinic acytylcholine receptor M1 [41,42], G protein coupled receptor PAC1 [43], protein kinase C [44,45], and low cholesterol [46].

In addition to the regulation of Aβ biosynthesis, Aβ clearance is also regulated. Clearance of Aβ combines several mechanisms, including the LXR/ABCA1/APOE pathway [47], degradation by endoproteases, reviewed by Nalivaeva *et al*. 2008 [48], transport across the blood-brain barrier involving RAGE and LRP1 receptors, reviewed by Deane and Zlokovic [49], lymphatic drainage, reviewed by Weller *et al*. [50] and microglial uptake of Aβ [51,52]. Indirect regulation of Aβ clearance has also been reported, for example, modulation of neprilysin endoprotease by somatostatin receptor signaling [53], and enhanced Aβ proteolysis dependent on the ApoE isoform [54]. In addition, resveratrol, a polyphenol in red wine, has been proposed to enhance Aβ clearance via the proteasome [55].

Thus, it seems reasonable to anticipate numerous molecular targets capable of altering Aβ levels, and that at least some of the targets should be relevant to Aβ formation in the brain. The ideal target should have the potential for robust brain Aβ-lowering without toxicity, and characteristics that facilitate development of inhibitors. We therefore took the approach of direct screening of brain Aβ levels in novel mouse gene knock out (KO) strains, an approach that has the dual advantages of providing evidence both for target effectiveness in Aβ-lowering and for target safety. Given that even optimized drug molecules may not be capable of 100% ablation of target function, we were most interested in finding KO strains with 50% or more reduction in brain Aβ levels. A total of 1930 viable homozygous gene ablations, representing 1926 genes, were tested. Surprisingly, none of these gene ablations exhibited robust decreases in Aβ. In addition, we also evaluated GPR3 KO mice, recently proposed as an Aβ target [26], but found no overall effect on levels of brain Aβ. In contrast, significantly increased brain Aβ was detected in samples from two mouse KO strains corresponding to the proteins PIGZ and QPRT, respectively involved in GPI anchor biosynthesis and the kynurenine pathway of tryptophan degradation. Thus, while the mouse KO screen did not directly identify novel targets for lowering Aβ, it did suggest a limited number of biochemical pathways that might be significant for regulation of brain Aβ levels *in vivo*, and that gene ablations causing a robust effect on brain Aβ are rare.

## Results

### A screen of mouse KO strains for altered brain Aβ levels

**Figure 1:**
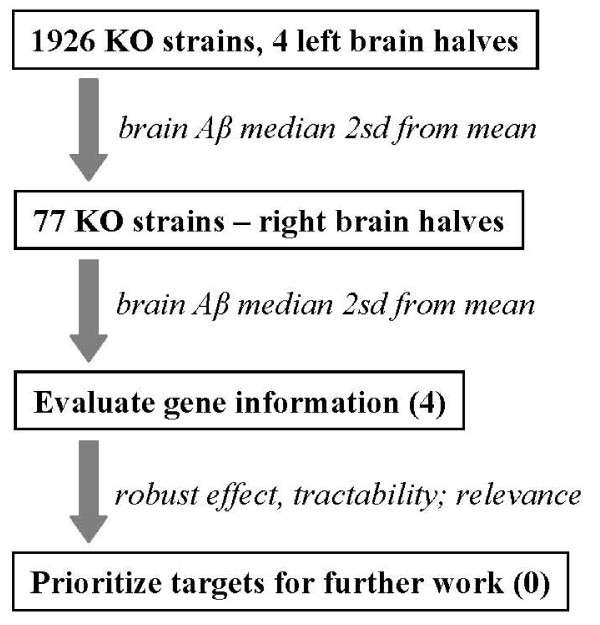
**Outline of screening tiers**. Aβ40 in four brain samples (sagittal left halves) from 1930 different mouse KO strains were determined. Of these, 77 KO strains were selected for further Aβ40 determinations, and in some cases Aβ42 determinations, of the corresponding right brain halves. The gene information for four of the KO strains is reported in the current study. Prioritization for possible drug discovery efforts was then based upon multiple considerations, including the robustness of Aβ inhibition, tractability of the target for small molecule inhibition, and relevance of the target to AD.

Mouse knock-out (KO) strains were generated over a period of four years, and brain samples were collected and assayed during this time on an ongoing basis. Figure 1 outlines the screening procedure. Using four homozygous individuals per KO strain, brain Aβ40 levels were determined using the left brain halves, and selected KO strains were chosen, based on potentially altered Aβ40 levels, for further Aβ determinations using the corresponding right brain halves. For KO strains in which Aβ median values were consistently altered, the identity of the gene KO was then revealed in order to make a decision on further studies. A summary of the 1926 genes by gene class is shown in Table 1, note that the sample is non-random and includes only strains in which the homozygous KO was viable.

**Table 1:** Summary of genes by class

Gene class	Number
Ion channel	85
DNA enzyme	9
Enzyme	307
G-Protein coupled receptor	89
Inhibitor	23
Kinase	193
Membrane and secreted	109
Membrane	304
Miscellaneous	40
Nuclear hormone receptor	4
Phosphodiesterase	18
Phosphatase	36
Protease	143
Putative secreted	57
Receptor associated protein	1
Secreted	317
Signaling	11
Cytoskeletal	2
Transporter	175
Transcription factor	3
Total	1926

**Figure 2:**
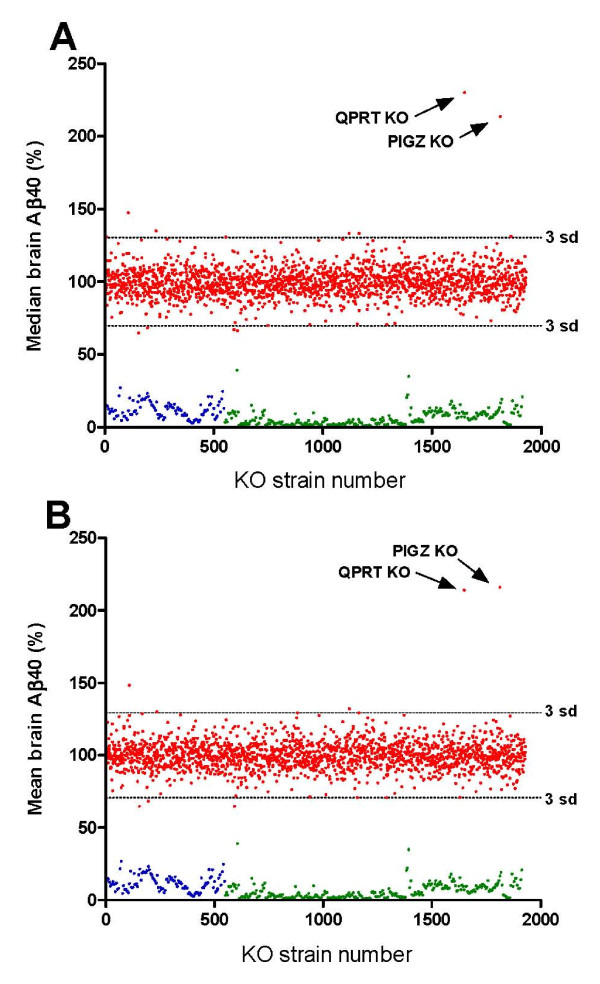
**Primary screen of Aβ40 levels**. Panel A - The median values of Aβ40 relative to baseline for each of 1930 KO strains is shown in red. Assay plate values for BACE1, BACE2 double KO brains are shown in blue, and values for Aβ1-14 synthetic peptide-blocked wild type brains are shown in green. The results for the PIGZ KO and the QPRT KO are indicated by arrows. Panel B - Same as panel A, except mean values of Aβ40 are plotted.

The results of the primary Aβ40 screen for 1930 different KO strains are illustrated in Figure 2. The data were well approximated by a normal distribution (Shapiro-Wilk's test p > 0.05) with standard deviation of 10%, indicating that a KO strain exhibiting a robust decrease of 50% would have been detected with 98% probability. The false positive error rate for observing a KO strain more than three standard deviations below the mean was estimated to be 0.13%. In spite of the high probability of detecting Aβ40 lowering, none of the novel 1930 KO strains appeared to lower Aβ40 by an amount that could be unequivocally distinguished from the general distribution. In contrast, samples from two KO strains exhibited approximately 100% increased Aβ40, which, at greater than 10 sd above baseline level, were far outside the general distribution (Figure 2). The false positive error rate associated with 10 sd is less than 6.7E-16. The genes corresponding to these two KO strains were PIGZ, encoding an enzyme required for 4th mannose side chain addition to the GPI protein anchor precursor, and QPRT, encoding quinolinate phosphoribosyltransferase, an enzyme of the kynurenine pathway of tryptophan degradation (see Discussion).

**Figure 3:**
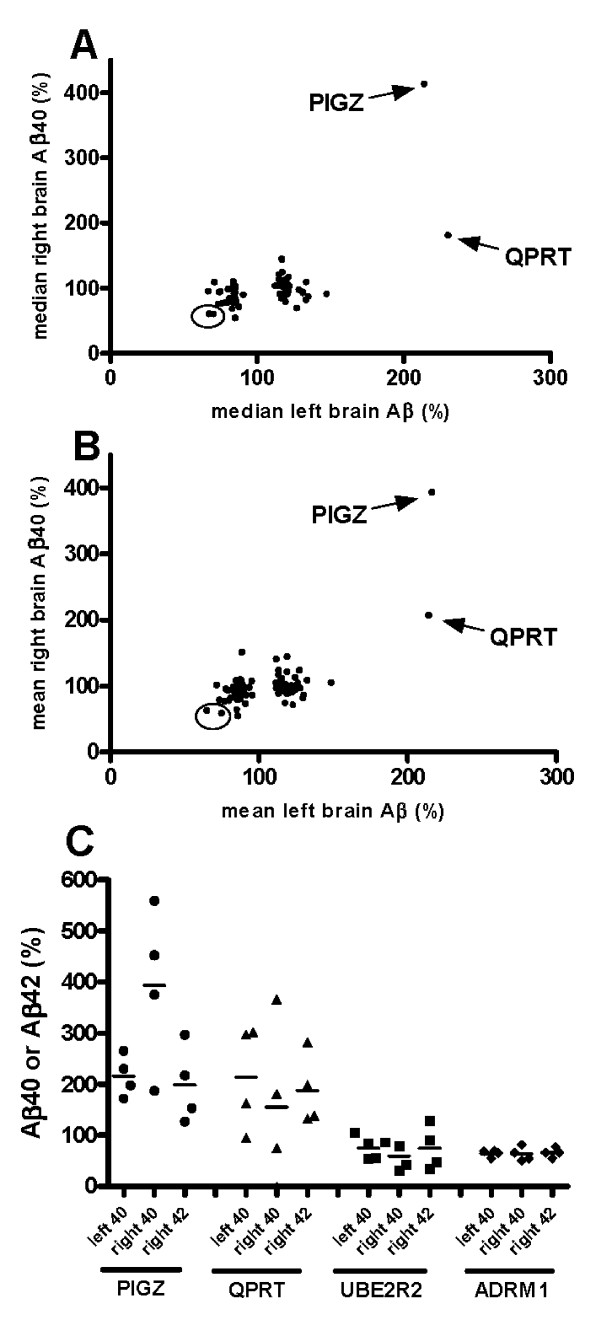
**Aβ levels in left and right brain halves of selected KO strains**. Panel A - Median values of Aβ40 in right brain halves were plotted against median values in left brain halves. An ellipse highlights the results for UBE2R2 and ADRM1. The results for the PIGZ KO and the QPRT KO are indicated by arrows. Panel B - Same as panel A, except that mean Aβ values were plotted. Panel C - Relative values of Aβ40 and Aβ42 from left and right brain halves in individual animals is shown for four named KO strains. Note that left and right are not directly comparable because different extraction and assays procedures were used (see Methods). Bars represent the mean value.

For 77 selected KO strains, particularly those exhibiting a median Aβ value more than 2 sd below control values, repeat Aβ40 assays, and in some cases Aβ42 assays, were carried out using the right brain half (Figure 3 panels A and B). For most of these 77 KO strains, the small changes in average Aβ40 levels were similar in magnitude to the assay variability, and it was therefore no surprise that very few of the results confirmed consistent changes of Aβ40 in the right brain halves. Consistent with this, there was no significant difference between the Aβ values in the right and left brain halves. (The mean difference was 3.23%, and a paired *t*-test yielded a *t*-value of 0.98, with 76 degrees of freedom, and a *P*-value of 0.33.) Nevertheless, the two KO strains that exhibited the greatest Aβ40-lowering in both left and right brain assays were UBE2R2 and ADRM1, both involved in the ubiquitin/proteasome system (UPS, see Discussion). In contrast, the robust average Aβ40 increases exhibited in the PIGZ KO and QPRT KO were readily confirmed in the right brain halves (Figure 3 panels A and B). Changes in mean Aβ42 were consistent with the changes in Aβ40 for the PIGZ, QPRT, UBE2R2 and ADRM1 KO mice (Figure 3 panel C). In the case of the ADRM1 KO, all the Aβ values were tightly grouped at about 30% lowering, whereas PIGZ, QPRT and UBE2R2 KO strains exhibited considerable individual variability in Aβ levels. For PIGZ, the difference in Aβ40 values between the left and right brain assays possibly reflected the different extraction procedures used; CHAPS extraction for the left and guanidine/SPE extraction for the right half (see Materials and Methods). However, this difference was not statistically significant (Table 2).

**Table 2:** Paired *t*-tests conducted on UBE2R2, ADRM1, PIGZ and QPRT KO strains

Left vs. right comparisons	Mean difference	*t*-value (degrees of freedom)	*P*-value
UBE2R2 KO	15.44	2.40 (3)	0.0957
ADRM1 KO	1.64	0.24 (3)	0.8286
PIGZ KO	-177	-2.48 (3)	0.0894
QPRT KO	58.64	0.72 (3)	0.5229

### Ablation of GPR3 did not affect total brain Aβ levels

**Figure 4:**
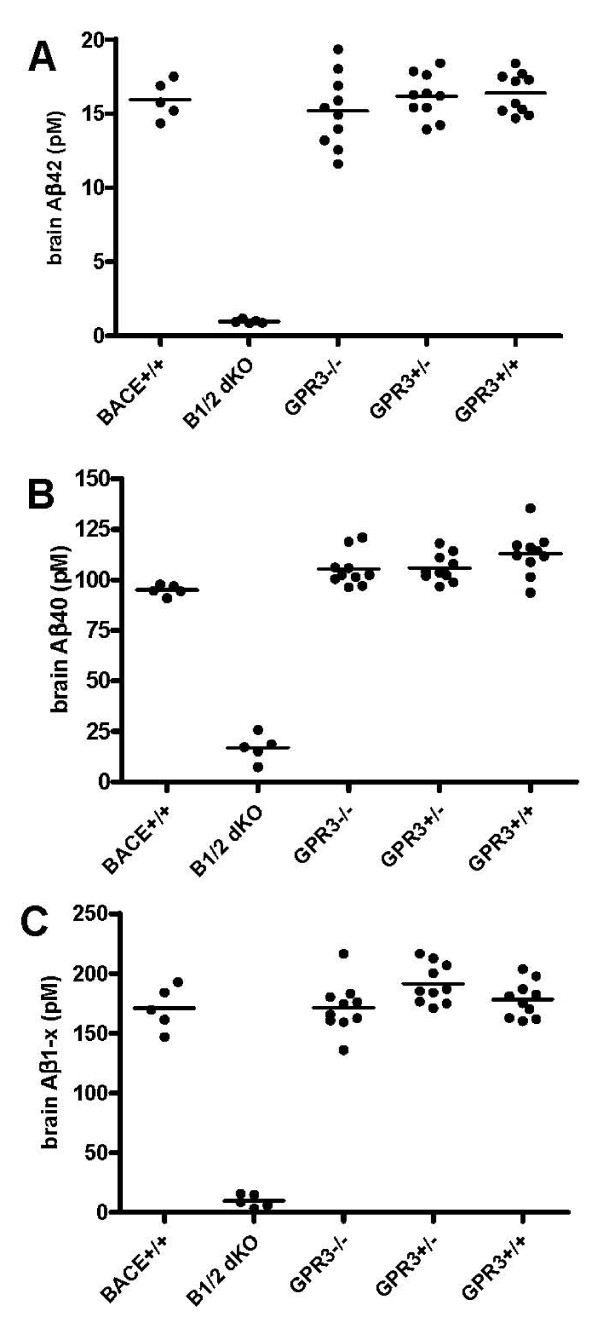
**Effect of GPR3 KO on brain Aβ levels in young mice**. Panel A - Aβ42 was assayed in brain extracts from ten GPR3 wild type (GPR3+/+), ten heterozygous (GPR3+/-), and ten homozygous GPR3 KO (GPR3-/-) mice. Brain Aβ42 was also determined in BACE1, BACE2 homozygous double KO (B1/2 dKO) and wild type (BACE+/+) control mice. Bars represent the mean values. Panel B - the same brain extracts were assayed for Aβ40. Panel C - the same brain extracts were assayed for Aβ1-x.

**Figure 5:**
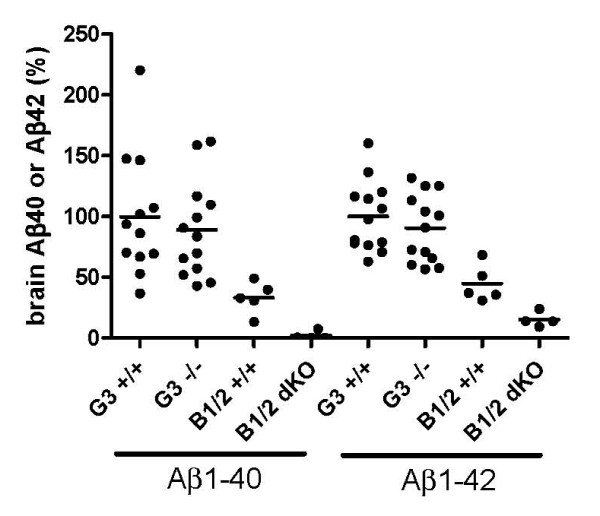
**Effect of GPR3 KO on brain Aβ levels in aged mice**. Aβ40 and Aβ42 were determined in one year aged mice for 13 GPR3 wild type (G3+/+) and 13 homozygous GPR3 KO (G3-/-) mice. For comparison, Aβ40 and Aβ42 for five young wild type (B1/2+/+) and four young BACE1, BACE2 homozygous double KO (B1/2 dKO) are shown. Values are expressed as percentage of mean value in the GPR3 wild type mice. One individual GPR3 wild type mouse with a value of 624% was excluded from the analysis.

Deletion of the orphan G protein coupled receptor GPR3 has recently been reported to decrease hippocampal Aβ40 and Aβ42 in APP transgenic mice by a mechanism that involves the regulation of γ-secretase [26]. We engineered a GPR3 KO strain and carried out Aβ assays using extracts from sagittal brain halves (see Materials and Methods). Young mice showed no significant differences in brain Aβ42, Aβ40 or Aβ1-x levels between mice carrying the homozygous or heterozygous KO genotypes and homozygous wild type siblings (Figure 4). Mice aged one year also showed no significant difference in brain Aβ40 and Aβ42 levels between the GPR3 KO and wild type siblings, although they exhibited a greater degree of individual variation in Aβ40 and Aβ42 levels that made it more difficult to detect small differences in the mean values (Figure 5). The results of significance testing for the different Aβ species in young and old animals are shown in Table 3.

**Table 3:** Comparisons between GPR3 KO and wild type animals using independent samples *t*-tests

Comparisons	Mean Difference	*t*-value (degrees of freedom)	*P*-value
Aβ40 young -/- vs +/+	-7.69	-1.76 (18)	0.0955
Aβ40 young +/- vs +/+	-6.99	-1.71 (18)	0.1051
Aβ42 young -/- vs +/+	-1.21	-1.37 (18)	0.1890
Aβ42 young +/- vs +/+	-0.22	-0.34 (18)	0.7374
Aβ1-x young -/- vs +/+	-6.70	-0.82 (18)	0.4211
Aβ1-x young +/- vs +/+	13.34	1.90 (18)	0.0737
Aβ40 old -/- vs +/+	-51.56	-1.17 (24)	0.2519
Aβ42 old -/- vs +/+	-9.55	-0.86 (24)	0.3998

## Discussion

To identify novel molecular targets relevant to regulation of Aβ levels in the brain, we screened viable mouse KO strains for decreased brain Aβ40. A total of 1930 different gene ablations giving rise to viable homozygous mice were evaluated. Two of the strains, PIGZ KO and QPRT KO, showed an unequivocal increase of Aβ40 and Aβ42 in the screening samples, whereas changes in Aβ were less obvious for the other KO strains. The two KO strains showing the lowest values of Aβ40 and Aβ42 in the screen, UBE2R2 and ADRM1, were of uncertain significance given the small number of samples tested.

Given the wide variety of mechanisms and proteins that have been reported to affect Aβ, and the relatively large number of gene KO strains tested, it was surprising that we did not identify a single new gene KO strain that caused a robust (≥50%) decrease in Aβ. A combination of several reasons may account for this. First, the choice of gene KO strains entering the screen was based on the 'druggable genome', which mostly limited the KO strains to proteins in gene families known to interact with small molecules. Second, the gene KO strains entering the screen were limited to those strains that resulted in viable homozygous adult mice. Approximately one third of the gene KO constructs made were homozygous embryonic lethal, and were consequently not included in the brain Aβ screen. Therefore, effective Aβ-lowering targets such as presenilin 1, for which genetic ablation is deleterious, would not have been tested in this screen. Third, functional redundancy, e.g. the genes encoding the Aph1B and Aph1C subunits of γ-secretase [56], could have obscured any effects of single gene ablations. There is also the possibility of developmental compensation, in which alternative pathways functionally substitute for the missing gene, thus restoring Aβ levels in the adult. Fourth, inbred mice have been shown to express significantly different levels of brain Aβ [57], raising the possibility that genetic changes in these mice may have obscured the function of some genes (epistasis). Fifth, there is the possibility that some genes may affect Aβ levels only in older mice, and therefore the role of these putative genes would not have been apparent at the age of three months when our mice were harvested. Sixth the ability of the screen to detect changes in Aβ was limited by the intrinsic variability in Aβ combined with the small group size, which in most cases was equal to four homozygous KO animals. This limitation to four animals per KO strain was necessary because of the resource and time constraints of producing and maintaining multiple KO mouse colonies, and the use of most of the available KO mice for other phenotypic and biochemical tests not reported here. Nevertheless the statistical power of the screen was favorable. Based on the good fit of the data to the normal distribution (Shapiro-Wilk's test p > 0.05), the false negative error rate (Type II error) was found to be 1.7% for detection of a KO strain with 50% lowering of Aβ, and 5.99E-9 for a KO strain with 85% lowering of Aβ, as observed in the BACE1 KO samples. Thus, there was a high probability for detection of any KO strains robustly lowering Aβ. In addition, the most practical Aβ-lowering targets should have the potential to lower Aβ by a robust and substantial amount, and thus, for the purpose of identifying the most practical targets, a low group size could be tolerated. The false positive error rate (Type I error) for a KO strain more than 3 sd below the mean was 0.13%, and for 10 sd above the mean was negligible at less than 6.7E-16. Thus, the results for the two KO strains, UBE2R2 and ADRM1, which exhibited the lowest brain Aβ levels around three standard deviations below the mean, could have been due to chance, whereas the Aβ increases in the PIGZ and QPRT KO strains were very unlikely due to chance. Finally, despite the possible limitations discussed above, it is hard to escape the conclusion that molecular targets capable of robust Aβ lowering in the relevant context of brain are rare.

Brain Aβ40, Aβ42 and Aβ1-x levels in GPR3 KO mice were evaluated more thoroughly by using a larger number of mice. No changes in brain Aβ42, Aβ40 or Aβ1-x were detected in sagittal brain halves from these mice. This contrasts with the results reported by Thathiah *et al*. [26] in which ca. 50% lowering of Aβ40 and Aβ42 was observed in APP-transgenic GPR3 KO mice. There are two noteworthy experimental differences between the two studies, first, we assayed endogenous mouse Aβ, not transgenic human Aβ, and second, we used sagittal brain halves not hippocampal sections. Unfortunately, our analysis did not extend to hippocampal sections, which constitutes only a small fraction of total brain. GPR3 is expressed at high levels in the cortex, which, like hippocampus, is relevant to AD. Thus, evaluation of Aβ in the hippocampus of non-APP GPR3 KO and in the cortex and/or whole brain of APP transgenic GPR3 KO would be interesting.

Two gene ablations, corresponding to the PIGZ and QPRT enzymes, exhibited significantly increased Aβ40 and Aβ42 in the screening samples. While further substantiation of the results for PIGZ and QPRT using larger groups of homozygous KO mice is clearly desirable, plausible molecular mechanisms for increased Aβ can be proposed. PIGZ, also known as SMP3, is a mannosyl transferase that catalyses addition of a fourth side chain mannose to the glycosylphosphatidylinositol (GPI) protein anchor precursor [58,59]. In cell cultures, GPI anchored proteins are necessary for Aβ synthesis [20], and targeting of an artificial BACE1-GPI chimera to lipid rafts greatly increases Aβ production [22], although targeting of BACE1 to lipid rafts is not necessary for Aβ synthesis [60]. Thus, in cell culture, a connection between GPI anchor metabolism and Aβ levels is well established. An effect of PIGZ on brain Aβ would extend these conclusions to a relevant organ *in vivo*, and further raises the possibility that the fourth mannose residue plays a specific role in Aβ metabolism. QPRT is the enzyme responsible for quinolinic acid turnover in the kynurenine pathway of tryptophan degradation [61], and therefore ablation of this gene would be expected to increase quinolinic acid levels. Increased quinolinic acid has been reported in AD brain [62,63], and treatment of primary neuronal cultures with quinolinic acid has been reported to increase cell death and oxidative stress [64]. The association of oxidative stress with increased BACE1 activity and Aβ production has been widely substantiated in AD [10,15,65-69], raising the possibility of a mechanistic connection between quinolinic acid and Aβ through activation of BACE1 by oxidative stress. In addition, quinolinic acid has been reported to increase APP expression in rat brain, which could contribute to increased Aβ production [70].

The two KO strains for which the lowest values of Aβ40 and Aβ42 (ca. 30% lowering) were observed in our screen corresponded to the UPS proteins Adrm1 and Ube2R2. Adrm1, also known as hRpn13, associates with the proteasome 19S regulatory particle, and is required for recruitment of the Uch37 deubiquitinating enzyme to the proteasome [71,72]. Ube2R2 (sequence NM-017811) is a ubiquitin conjugating enzyme. Decreased expression of several other ubiquitin conjugating enzymes has been reported to decrease Aβ production in cell culture [73]. The UPS has multiple potential roles in AD in addition to possible regulation of Aβ levels, as recently reviewed in detail by Upadhya and Hegde [74]. Possible mechanisms of proteasomal regulation of Aβ include resveratrol-activated clearance of Aβ [55], and competition with γ-secretase for APP processing [27]. Thus, an intriguing possibility is that selective inhibition of specific sub-pathways of the UPS might decrease brain Aβ levels by both biosynthetic and clearance mechanisms. However, from a drug discovery perspective, this would carry the risk of further exacerbating the already defective proteasome activity prevalent in AD thought to result from the accumulation of toxic Aβ and tau aggregates. Furthermore, assuming that maximal inhibition of Adrm1 or Ube2R2 would elicit only a 30% decrease in brain Aβ, even the effect of an inhibitor with ideal drug properties would be limited, and the expected small changes in Aβ difficult to quantify.

## Conclusions

Gene ablations that have a robust effect on brain Aβ appear to be rare, at a rate of approximately one in a thousand of the genes reported here. However, several pathways including GPI anchor metabolism, the kynurenine pathway of tryptophan degradation, and the UPS may be worth further evaluation for their roles in brain Aβ regulation.

## Methods

### Mouse KO strains and brain samples

Experimental procedures with mice were authorized by, and in compliance with, the Lexicon Pharmaceuticals Animal Care and Use Committee, and the Bristol-Myers Squibb Animal Care and Use Committee. The BACE1, BACE2 and GPR3 KO strains were provided by Lexicon Pharmaceuticals under the terms of the LexVision^® ^Database and Collaboration Agreement. The BACE1/BACE2 double KO obtained by intercrossing the BACE1 and BACE2 KO strains has been described previously [75]. The GPR3 KO was made by targeted homologous recombination in mouse strain 129SvEv^Brd^-derived embryonic stem cells, using a targeting construct containing a bGeo/puromycin selection cassette to remove 1,023 nucleotides encompassing the entire amino acid coding region in the single exon of this gene (see NCBI nucleotide reference sequence NM_008154 for GPR3). Recombination in ES cells was confirmed by Southern analysis. Chimeric mice were bred with C57BL/6J albino mice to generate F1 heterozygous animals. The F1 mice were intercrossed, and the genotypes of F2 progeny were determined by Southern analysis. The observed genotype frequencies, 20 wild type, 33 heterozygous and 15 homozygous mice, were not significantly different from the Mendelian segregation ratios expected for a viable allele. The genotypes of further F2 progeny were determined by PCR of tail or ear DNA using the DNA primer 5'-GAATTAAGCCCTGGTGGACCTA, corresponding to sequence adjacent to the GPR3 deletion, in combination with the primer 5'-GTTGCCCTTCACTGTCTACTGC, corresponding to deleted GPR3 sequence, to detect a 286 nucleotide product from the wild type GPR3 allele, or in combination with the primer 5'-GCAGCGCATCGCCTTCTATC, derived from the neo marker gene, to detect a 208 nucleotide product from the GPR3 KO allele. For the GPR3 KO studies, animals were either 3 months old 'young' or 1 year old 'aged' at the time of harvesting. The other 1930 KO strains, corresponding to 1926 different genes, including the PIGZ, QPRT, and ADRM1 KO strains derived from the OmniBank^® ^gene trap library, and the UBE2R2 KO strain made by targeted homologous recombination, were made by Lexicon Pharmaceuticals as part of its Genome5000™ program [76]. A summary of the 1926 genes by gene class is shown in Table 1.

### Preparation and storage of brain samples

For the primary screen of brain Aβ levels, four mice from each KO strain were euthanized at three months of age by CO_2 _asphyxiation and sagittal brain halves lacking cerebellum were frozen and stored at -80°C. Samples were shipped on dry ice from Lexicon Pharmaceuticals to Bristol-Myers Squibb as they became available over a period of four years. Because the availability of individual mice occurred with varied timing, and because multiple different KO strains were in production simultaneously, the order of acquisition of brain samples was variable. This resulted in the four samples from any given KO strain arriving at different times, and thus the Aβ ELISA being carried out usually on different assay plates and different assay dates for each sample of a given KO strain. The Aβ primary screen values obtained even for a given gene KO therefore represented essentially all of the sources of variability to which the data were subject.

### Aβ sample preparation and ELISA assays

The assay used for the primary screen of all brain samples has been partly described previously [75]. Frozen left brain halves were thawed and homogenized at a concentration of 4 ml/g in ice cold 2% CHAPS, 20 mM Tris-Cl pH 7.7, in the presence of complete protease inhibitor cocktail (Roche Diagnostics, Mannheim, Germany). Homogenization was carried out by agitation in 2.0 ml polypropylene tubes containing a steel bead (5 mm diameter) for 4 min. at maximum power using the TissueLyzer (Qiagen). Homogenates were centrifuged at 21,000 × g at 4°C for 30 min. Supernatant from the centrifugation was stored frozen at -80°C, and thawed immediately before use in the ELISA. ELISA to quantify Aβ was carried out in 96-well format using three wells per sample. The capture antibody was the Aβ40 C-terminal end-specific monoclonal antibody TSD9S3.2 (Bristol-Myers Squibb), and detection was carried out using peroxidase conjugated monoclonal antibody 252Q6 (Invitrogen), specific for the N-terminal region of mouse Aβ. To confirm results for selected brain samples, the corresponding frozen right brain halves were thawed and homogenized in CHAPS buffer for assay of Aβ40, as described above. Alternatively, right brain halves were homogenized at a concentration of 10 ml/g in 6M guanidine by agitation with a steel bead, as described above, centrifuged at 21,000 × g at 4°C for 60 min., and Aβ was concentrated from the supernatant by solid phase extraction using Oasis HLB cartridges (Waters) on a vacuum manifold as described [77]. Eluates in methanol/0.1% NH_4_OH containing Aβ from the solid phase extraction were dried in a rotary evaporator under vacuum and resuspended in phosphate buffered saline pH 7.4 containing 0.1% bovine serum albumin (Sigma cat. #A7030) and 0.1% Triton X-100 immediately before use in ELISA. Aβ40 was quantified by ELISA in triplicate wells as described above, and Aβ42 was quantified by ELISA using the monoclonal 252Q6 (Invitrogen) as capture antibody, and Aβ42 C-terminal end-specific monoclonal 565 peroxidase conjugate (Bristol-Myers Squibb) as the detection antibody. To monitor assay performance, the first 109 assay plates contained control wells of wild type and BACE dKO extracts. The value of z' ranged from 0.5 to 0.74 for these first plates, indicating that any samples containing decreased Aβ should be readily detectable [78]. To monitor assay performance without the need for BACE dKO animals, subsequent plates contained wild type extract control wells in which the detecting antibody incubation contained rat Aβ1-14 synthetic peptide (Anaspec) at a concentration of 1 μg/ml. All reagents, unless otherwise stated, were obtained from Sigma. The guanidine/solid phase extraction method, as described above, was also used for determination of Aβ42 and Aβ40 in the aged GPR3 KO study. For the young GPR3 KO animals, brain samples were homogenized in 0.2% diethanolamine, 50 mM NaCl and protease inhibitor cocktail (Roche) for Aβ42 and Aβ40 ELISAs as described above. The Aβ1-x assay utilized the combination of monoclonals 252Q6 and 4G8 (Covance). Assay results were calibrated using synthetic Aβ42 or Aβ40 peptides (Anaspec) and expressed as average pM concentration present in brain tissue prior to homogenization.

### Evaluation of Aβ results

To maintain consistency in the Aβ40 determinations between assay plates, the baseline value of Aβ40 was set equal to the mean value of all wells containing brain samples, excluding the BACE1/2 double KO or Aβ1-14 inhibited wells. Thus, the plate baseline generally depended on the mean value of 72 wells per plate. Alternative baseline values determined from known concentrations of synthetic Aβ40 peptides, or determined from pools of brain extracts from wild type mice, were found to be less consistent over time, and were therefore not used for evaluation of Aβ40 baseline levels. Median values of Aβ40 were calculated to avoid the potentially disproportionate effect of unusually high or low individual samples that could occur if using mean values, however, in the final analysis, use of mean or median values yielded the same outcome. Initially, calculations were carried out using data from a limited number of KO strains, and therefore some initial follow up assays were carried out for KO strains for which the median Aβ40 value was less than 2 sd from baseline.

## Authors' contributions

JHT directed the project for part of its duration, designed and optimized Aβ assay methods for mouse brain, carried out data analysis, and wrote the manuscript. X-AL, MWT and VG carried out Aβ assays and data analysis. JEM carried out data analysis and directed the project for part of its duration. SS designed and optimized Aβ assay methods for mouse brain. NB carried out brain dissection for the GPR3 KO studies. JC evaluated genes that were of potential interest. AM carried out statistical analysis. DLS directed initial stages of the project, data analysis and mouse husbandry. MH carried out mouse husbandry and brain dissection. CFA, TL and RSW provided strategic guidance and project coordination. All authors read and approved the manuscript.
